# Chronic Active Antibody-Mediated Rejection Is Associated With the Upregulation of Interstitial But Not Glomerular Transcripts

**DOI:** 10.3389/fimmu.2021.729558

**Published:** 2021-09-20

**Authors:** Andriy Trailin, Petra Mrazova, Petra Hruba, Ludek Voska, Eva Sticova, Antonij Slavcev, Marek Novotny, Matej Kocik, Ondrej Viklicky

**Affiliations:** ^1^Transplant Laboratory, Institute for Clinical and Experimental Medicine, Prague, Czechia; ^2^Department of Clinical and Transplant Pathology, Institute for Clinical and Experimental Medicine, Prague, Czechia; ^3^Department of Immunogenetics, Institute for Clinical and Experimental Medicine, Prague, Czechia; ^4^Department of Nephrology, Transplant Centre, Institute for Clinical and Experimental Medicine, Prague, Czechia; ^5^Institute of Physiology, 1^st^ Faculty of Medicine, Charles University, Prague, Czechia; ^6^Transplantation Surgery Department, Institute for Clinical and Experimental Medicine, Prague, Czechia

**Keywords:** kidney transplantation, antibody-mediated rejection, gene expression, laser capture microdissection, renal compartments

## Abstract

Molecular assessment of renal allografts has already been suggested in antibody-mediated rejection (ABMR), but little is known about the gene transcript patterns in particular renal compartments. We used laser capture microdissection coupled with quantitative RT-PCR to distinguish the transcript patterns in the glomeruli and tubulointerstitium of kidney allografts in sensitized retransplant recipients at high risk of ABMR. The expressions of 13 genes were quantified in biopsies with acute active ABMR, chronic active ABMR, acute tubular necrosis (ATN), and normal findings. The transcripts were either compartment specific (*TGFB1* in the glomeruli and *HAVCR1* and *IGHG1* in the tubulointerstitium), ABMR specific (*GNLY*), or follow-up specific (*CXCL10* and *CX3CR1*). The transcriptional profiles of early acute ABMR shared similarities with ATN. The transcripts of *CXCL10* and *TGFB1* increased in the glomeruli in both acute ABMR and chronic active ABMR. Chronic active ABMR was associated with the upregulation of most genes (*SH2D1B*, *CX3CR1*, *IGHG1*, *MS4A1*, *C5*, *CD46*, and *TGFB1*) in the tubulointerstitium. In this study, we show distinct gene expression patterns in specific renal compartments reflecting cellular infiltration observed by conventional histology. In comparison with active ABMR, chronic active ABMR is associated with increased transcripts of tubulointerstitial origin.

## Introduction

Both acute antibody-mediated rejection (ABMR) and chronic ABMR are the main risk factors for late renal allograft loss (1–3). Sensitization to donor human leukocyte antigen (HLA) represents the main risk factor for ABMR development (4–6). Molecular diagnostics has already been suggested to improve the diagnostic accuracy of ABMR by histological assessment (7–9). Recent molecular methods evaluate biopsy specimens irrespective of the cortex or medulla origin due to the proportion of the cortex having little influence on the molecular diagnosis of rejection (10), while conventional histological assessment relies specifically on the cortex. The topography of intrarenal transcripts is important in understanding the mechanisms of ABMR; however, it has not yet been systemically studied, mainly due to technical obstacles.

*In situ* hybridization (ISH) is a powerful technique for the identification of specific messenger RNA (mRNA) expressions within individual cells in tissue sections, providing insights into physiological processes and disease pathogenesis. However, this complex method is technically difficult and requires the precise optimization of many steps for each tissue examined and for each probe used. Although many improvements have been made, the main limitation of ISH remains its poor sensitivity (11, 12). Aside from the NanoString platform (13), laser capture microdissection (LCM), when combined with molecular techniques, has demonstrated the ability to detect gene transcripts from distinct renal architecture (14). Until now, transcriptomic data based on LCM in renal allografts are limited (15–17) due to excessive necessary workload and the limited availability of sufficient tissue to enable high-quality transcripts.

In this study, we used LCM to examine the compartment-specific expression patterns of selected genes in sensitized kidney transplant recipients who suffered from active and chronic active ABMR.

## Patients and Methods

### Patient’s Characteristics and Sample Collection

**Figure 1:**
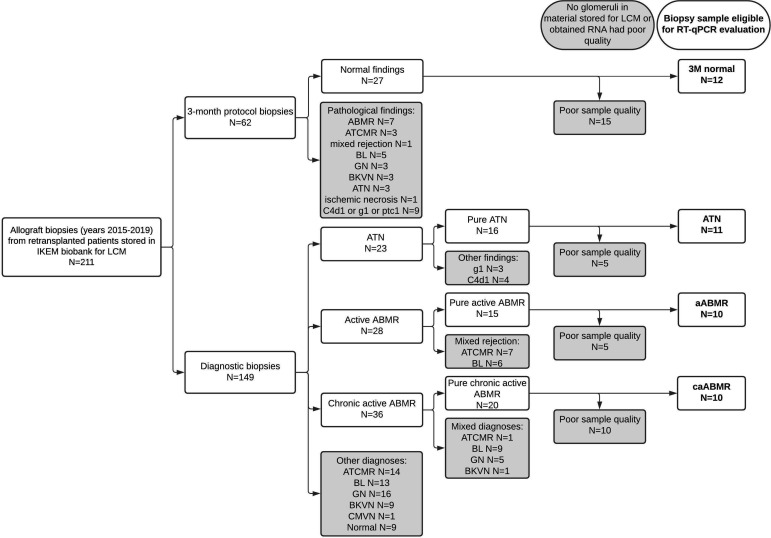
Flowchart of samples selection. Only samples with pure diagnosis of active ABMR, chronic active ABMR, ATN and normal findings in 3-month protocol biopsy, which had enough material for LCM and good RNA quality, were used for RT-qPCR form the biobank of biopsies from retransplanted patients. LCM, laser capture microdissection; ABMR, antibody-mediated rejection; ATCMR, active T-cell mediated rejection; BL, borderline lesions; GN, recurrence of glomerulonephritis; BKVN, BK virus nephropathy; CMVN, cytomegalovirus nephropathy; ATN, acute tubular necrosis.

A small portion (~2 mm) of all for-cause and 3-month (3M) protocol biopsies, performed from January 2015 until January 2019 in sensitized kidney transplant recipients who had undergone a second, third, or a fourth kidney transplantation, were embedded into Tissue-Tek^®^ O.C.T. Compound (Sakura Finetek USA, Inc., Torrance, CA, USA), snap frozen in liquid nitrogen immediately, and then stored at −80°C for future LCM and gene expression analyses (*N* = 211). We identified patients with active ABMR (aABMR, *N* = 28) and chronic active ABMR (caABMR, *N* = 36). Biopsies with coincidental T-cell-mediated rejection (TCMR), borderline lesions, polyomavirus nephropathy, or recurrent disease were excluded. Biopsies from patients with acute tubular necrosis (ATN) diagnosed early after transplantation (*N* = 23) and normal findings at 3M protocol biopsies (*N* = 27) served as controls. Samples with absence of the renal cortex or glomeruli and samples with insufficient RNA quantity were excluded. The final cohort consisted of 43 patients (aABMR, *N* = 10; caABMR, *N* = 10; early ATN, *N* = 11; and 3M normal findings, *N* = 12) (**Figure 1**).

The demographics and clinical characteristics of the patients are summarized in **Table 1**. Both aABMR and caABMR were defined according to the recent Banff classification (7). All patients from the ATN group displayed ATN along with mild chronic lesions. All stable patients from the control group were negative for donor-specific antibodies (DSA) and exhibited only a mild degree of arteriosclerosis, arteriolar hyalinosis, and interstitial fibrosis and tubular atrophy (IFTA) on biopsy. Detailed Banff scores of all the studied groups are given in **Supplementary Table S1**.

**Table 1:** Patients’ demographics and clinical characteristics according to the studied groups.

	ATN	Active ABMR	*p*-value	Normal	Chronic active ABMR	*p*-value
*N*	11	10		12	10	
Biopsy follow-up (POD)^a^	9 (6–48)	10 (5–144)	0.456	100 (84–110)	2,004 (231–4,607)	<0.001
Serum creatinine at biopsy (μmol/L)^a^	299.7 (137.0–754.5)	294.2 (65.8–806.4)	0.439	121.6 (62.2–134.6)	179.4 (142.8–376.7)	<0.001
Age (years)^a^	48 (39–74)	39 (26–64)	0.078	53 (35–68)	46 (27–60)	0.080
Male, *N* (%)	9 (82)	8 (80)	1.000	8 (67)	9 (90)	0.323
No. of transplants (second/third/fourth)	9/2/0	7/2/1	0.547	10/2/0	6/3/1	0.361
Dialysis vintage (months)^a,b^	58 (37–238)	58 (26–154)	0.778	55 (11–103)	58 (13–232)	0.872
PRA max, *N* (%)^a^	10 (0–90)	75 (20–98)	0.022	19 (2–96)	80 (2–98)	0.050
Anti-HLA class I and/or II positive, *N* (%)^c^	10 (91)	10 (100)	1.000	8 (44)	10 (100)	0.096
DSA class I and/or II positive, *N* (%)^c^	6 (55)	9 (90)	0.149	0 (0)	5 (50)	0.01
Anti-MICA positive, *N* (%)^c^	0 (0)	3 (30)	0.090	1 (8.3)	3 (33.3)	0.272
Induction, *N*			0.543			0.244
None	0	0		0	1	
Basiliximab/daclizumab	1	0		0	1	
ATG	7	8		12	7	
ATG, rituximab	3	2		0	1	
Donors’ age (years)^a^	63 (39–73)	50 (35–58)	0.024	55 (36–65)	52 (7–65)	0.372
Donor type (D), *N* (%)	11 (100)	10 (100)	1.000	11 (92)	9 (90)	1.000
Male, *N* (%)	5 (46)	6 (60)	0.670	8 (67)	7 (70)	1.000
ECD, *N* (%)	6 (55)	3 (33)	0.387	4 (33.3)	6 (66.7)	0.198
CIT (h)^a^	14.3 (9.1–23.2)	16.2 (10.3–20.6)	0.573	14.9 (3.4–20.5)	18.4 (0–23)	0.118
Mismatches^a^	2 (0–6)	4 (2–6)	0.029	3 (0–5)	4.5 (2–6)	0.008

ABMR, antibody-mediated rejection; PRA, panel-reactive antibodies; HLA, human leukocyte antigen; DSA, donor-specific antibodies; MICA, major histocompatibility complex class I chain-related antigen A; ATG, anti-thymocyte globulin; D, deceased; ECD, expanded criteria donor; CIT, cold ischemia time.

a Data are presented as median (minimum–maximum).

b Total time on dialysis.

c At the time of biopsy.

The present research was carried out in accordance with ethical standards and was approved by the Ethics Committee of the Institute for Clinical and Experimental Medicine in Prague (no. A 13-02-01). All patients gave written informed consent for participation in the study.

### Laser Capture Microdissection

All biopsies were performed using a semiautomatic biopsy gun with 16-G needle (Möller Medical GmbH, Fulda, Germany) guided by ultrasound. A 2-mm piece of biopsy tissue was embedded into Tissue-Tek, snap frozen with liquid nitrogen, and then kept at −80°C for future LCM.

Sections 10 μm thick were cut on a cryomicrotome (Leica CM1950, Wetzlar, Germany) at −20°C. Up to five sections were mounted onto each RNase-free MMI Membrane Slide (MMI AG, Glattbrugg, Switzerland). The slides were stored inside the cryochamber until the last section was cut and then, without defrosting, the sections were immediately fixed in ice-cold 96% ethanol for 45 s. After removal of Tissue-Tek in ice-cold 70% ethanol, the sections were rehydrated in ice-cold RNase-free water, stained in hematoxylin for 30 s, rinsed in RNase-free water, stained by eosin for 45 s (H&E staining kit for LCM; MMI AG, Glattbrugg, Switzerland), rinsed again in RNase-free water, and then dehydrated in two portions of ice-cold 100% ethanol for 45 and 60 s. The slides were then air dried, inverted, and placed onto a glass object slide for protection against contamination. Then, the sections were immediately microdissected using the inverted Olympus IX70 microscope (Olympus, Hamburg, Germany) coupled with the microdissection system MMI CellCut^®^ (Molecular Machines&Industries, Zürich, Switzerland), which uses low-damage UV laser.

All available glomeruli (except those with global glomerulosclerosis) were delineated excluding Bowman’s capsule. Tubulointerstitial areas, which included the cortical labyrinth, medullary rays, and the surrounding interstitium with peritubular capillaries, were outlined as well. Selected compartments were dissected by a laser microbeam through the objective and collected onto the adhesive lid of 0.5ml MMI isolation caps (MMI AG, Glattbrugg, Switzerland). For each biopsy, a median of 61 glomerular cross-sections (min–max = 11–173), corresponding to a median area of 1.36 mm^2^ (min–max = 0.34–4.00 mm^2^), and the cortical tubulointerstitium (TI) with a median area of 7.50 mm^2^ (min–max = 1.93–17.10 mm^2^) were collected. All procedures were performed under strict RNase-free conditions. The microdissection time did not exceed 30 min to avoid the action of RNases (as was established in preliminary experiments).

### RNA Extraction, cDNA Synthesis, and Real-Time PCR

The microdissected tissue samples were immediately homogenized by vortexing in RLT lysis buffer on the basis of guanidine thiocyanate (RNeasy Micro Kit, Qiagen, Hilden, Germany), containing 1% of β-mercaptoethanol (Sigma-Aldrich, Taufkirchen, Germany), and stored at −20°C until RNA isolation. For each patient, all dissected glomeruli or TI were pooled for RNA extraction. Total RNA was isolated using the RNeasy Micro Kit (Qiagen, Hilden, Germany) according to the manufacturer’s instructions. Reverse transcription was performed using SuperScript II reverse transcriptase (Invitrogen, Thermo Fisher Scientific, Waltham, MA, USA). Only the samples with Ct_GAPDH_ values <32 were included.

Due to the limited amount of material obtained by LCM, which was not suitable for whole-transcriptome analysis, gene expression profiling using a custom-made TaqMan low-density array (TLDA) cards (Applied Biosystems, Thermo Fisher Scientific, Waltham, MA, USA) enabling the measurement of 16 genes for eight samples per one card was used. Fourteen genes of interest were selected for expression profiling based on literature data regarding their implications in cellular immunity, humoral immunity, and injury/inflammatory response, plus glyceradyhyde-3-phosphate dehydrogenase (*GAPDH*) as the endogenous control and Nephrosis 2, Idiopathic, Steroid-Resistant, Podocin (*NPHS2*) as the gene expressed mainly in the glomeruli (**Supplementary Table S2**). All genes were measured in triplicate. Real-time quantitative reverse transcription PCR (RT-qPCR) amplification was performed on an ABI Prism^®^ 7900 H.T. Sequence Detection system (Applied Biosystems, Thermo Fisher Scientific, Waltham, MA, USA) using TaqMan Fast Advanced master mix (Applied Biosystems, Thermo Fisher Scientific, Waltham, MA, USA). The 2^−ΔΔCt^ method with GAPDH as an internal standard was used for quantification of the target gene expression. As a calibrator, one sample of TI from samples with normal histological findings at the 3-month protocol biopsy with good expression profiles of all the target genes was chosen. Gene expression data were normalized to *GAPDH* and relative to the calibrator sample (RQ manager 1.2. software for automated data analysis; Applied Biosystems, Thermo Fisher Scientific, Waltham, MA, USA), log2 transformed, and presented as fold change (FC) values. As the *TNFRSF17* gene was detectable in less than 15% of samples, it was excluded from all further analyses. As a control of sampling accuracy, glomerulus-specific *NPHS2* (podocin) was used (**Supplementary Figure S1**).

### Statistical Analysis

Differences in the gene expression or clinical parameters between groups were analyzed using the Mann–Whitney test or the chi-square test, where appropriate. Spearman’s rank correlation tests were performed for correlation analyses. Statistical analyses were performed using SPSS v.20.0 (SPSS, Inc., Chicago, IL, USA) and GraphPad Prism 5 v.5.03 for Windows (GraphPad Software, San Diego, CA, USA). Statistical significance was set at *p* < 0.05. Data are presented as median (minimum–maximum).

## Results

### Gene Expression Upregulation in ABMR

In this study, we evaluated transcripts thought to be associated with alloimmune response during ABMR (**Supplementary Table S2**). Initially, we compared the transcript expression patterns in the glomeruli and TI during ABMR and in controls, either samples from ATN as comparator for acute active ABMR or samples from normal findings as comparator for chronic active ABMR (**Supplementary Figure S2**).

The glomeruli in aABMR showed upregulation of the chemokine *CXCL10* compared to ATN, while the TI in aABMR exhibited increased *IFNG* signals (**Supplementary Figure S2A**). In caABMR, the intrarenal gene expressions of *GNLY*, *CX3CR1*, *TGFB1*, and *CXCL10* were upregulated in both the glomeruli and TI compared to normal findings at 3 months (**Supplementary Figure S2A**). The glomeruli of caABMR biopsies showed the upregulation of *CD14* and *IFNG* compared to normal control samples, whereas higher expressions of *SH2D1B* and *IRF4* were observed in the TI (**Supplementary Figure S2B**).

### Differences in Early Active ABMR and Chronic Active ABMR

**Figure 2:**
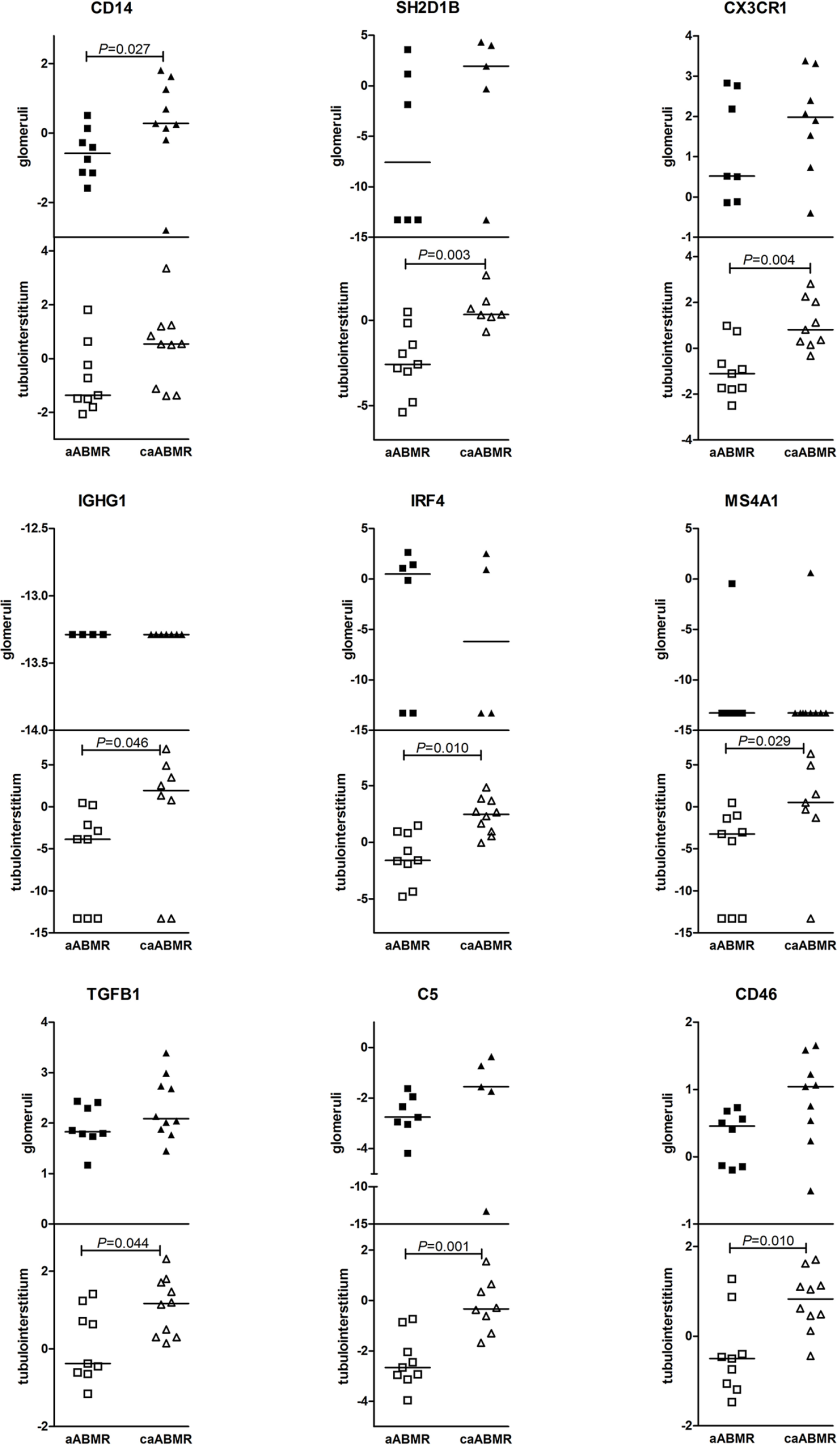
Differences in gene expression (log2 FC) between active ABMR (aABMR) and chronic active ABMR (caABMR) in glomeruli (dark marks) and tubulointerstitium (empty marks). Differences are calculated by Mann-Whitney test.

Chronic active ABMR differed from early active ABMR mainly in TI-associated transcript levels (**Figure 2**). Only one transcript, *CD14*, showed significant upregulation in the glomerular compartment of caABMR samples compared with aABMR (*p* = 0.027). The expressions of *CX3CR1*, *SH2D1B*, *IGHG1*, *IRF4*, and *MS4A1*, the complement-associated transcripts *C5* and *CD46*, and the *TGFB1* transcript were significantly upregulated in the TI in chronic active ABMR compared to active ABMR samples (**Figure 2**).

Therefore, genes associated with immune response were markedly upregulated in chronic ABMR compared to acute ABMR, especially in the interstitium, which suggests enhanced inflammation and fibrogenesis. This finding also corresponds well to conventional histology describing a higher total inflammation score. There was a strong correlation between the expressions of *CX3CR1*, *IFNG*, *IRF4*, and *TGFB1* in the TI and the Banff total inflammation score (*p* < 0.001; **Supplementary Table S3**).

### Different Gene Expression Patterns in Renal Compartments

**Figure 3:**
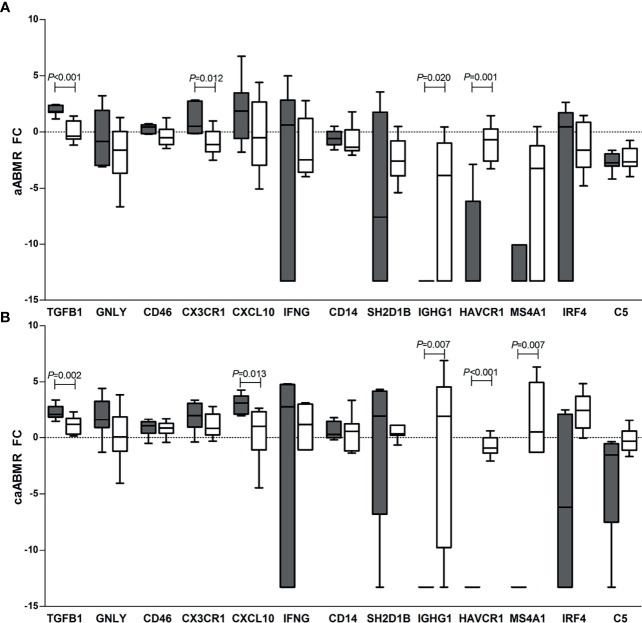
Differences in gene expression (log2 FC) between glomeruli (G, dark bars) and tubulointerstitium (TI, empty bars) in the groups with: **(A)** active ABMR (aABMR, n=10), and **(B)** chronic active ABMR (caABMR, n=10). Boxes extends from the 25th to 75th percentiles, lines in the middle of the box shows median and whiskers indicate the highest and lowest value within inner fences (Tukey whiskers). Differences are calculated by Mann-Whitney test.

Finally, we compared the gene expression patterns between the glomeruli and TI in all groups (**Supplementary Figure S3** and **Figure 3**). Regardless of the diagnosis, the *TGFB1* transcript was more highly expressed in the glomeruli while *IGHG1* and *HAVCR1* in the TI in all samples.

Non-rejection samples from ATN and normal findings exhibited higher glomerular expressions of *GNLY*, *CXCL10*, *CX3CR1*, and *CD46*, while there were higher TI expressions of *IGHG1*, *MS4A1*, *IRF4*, and *C5* (**Supplementary Figure S2**). In active ABMR samples, a higher glomerular expression of *CX3CR1* was described, while the upregulation of *IGHG1* was observed in the TI. In chronic active ABMR, *CXCL10* was more highly expressed in the glomeruli, while *MS4A1* and *IRF4* showed higher expressions in the TI (**Figure 3**).

## Discussion

The expressions of many genes of native kidney are compartment specific (18, 19). The gene expression patterns in inflamed kidneys during various kidney diseases or allograft rejection are supposed to correspond to the cellular infiltration observed in conventional histology. Despite current achievements in molecular diagnostics in kidney transplantation, little is known about renal compartment gene expression patterns in transplant pathologies. In this study, we used LCM to distinguish the gene expression patterns in the glomeruli and the tubulointerstitial area of kidney allografts in sensitized kidney retransplant recipients who were at high risk of ABMR development.

We found different expressions of transcripts associated with innate and adaptive immune responses in the glomerular and tubulointerstitial compartments. While the expressions of some genes associated with cellular infiltrates were increased in the glomeruli, others predominated in the TI. Contradictory data on gene expression patterns in various kidney graft compartments have been reported so far, mainly due to less advanced techniques and various diagnoses in stable grafts and during rejection. Of note is that strong correlations were reported between chromogenic ISH signals for chemokine transcripts and gene expression levels using the NanoString platform (13). Compared with the NanoString technology, RNA ISH and LCM coupled with RT-qPCR allow precisely assessing sites of gene expression. Previously, several studies have applied LCM coupled with RT-qPCR to evaluate the compartment-specific patterns of gene expression for ACE (20, 21), ion channels (22), cytokines (23, 24), and CD molecules (25, 26). The NanoString^®^ nCounter^®^ gene expression system has been recently shown to evaluate archival formalin‐fixed paraffin‐embedded tissues (27, 28). This technology allows gene expression analysis to be performed on the same tissue assessed with histology, permitting direct molecular–histologic correlations (29). Although this technology enables measuring up to 800 selected probes, its classification accuracy for kidney graft rejection based on formalin-fixed samples has not been validated in larger cohorts yet.

The antibody–endothelial interaction is associated with cellular infiltration of the glomeruli and peritubular capillaries. The Banff criteria for ABMR include microvascular inflammation (MVI) characterized by glomerulitis (g) and/or peritubular capillarities (ptc). Glomerular infiltration with monocyte/macrophages or natural killer (NK) cells has been frequently reported in ABMR (30–33), while the tubulointerstitial area was reported to be infiltrated to a less extent (30). However, in chronic active ABMR, the glomeruli displayed fewer T- and B-cell infiltrates than did the TI (34). B-cell aggregates in the TI were frequently observed as well, which is consistent with our findings describing the B-cell-related transcripts *IGHG1* and *MS4A1* to be upregulated in the TI in chronic active ABMR.

In our study, the transcriptional profile of active ABMR was found to be similar to ATN. Clearly, 8 of the 11 patients with ATN had DSA at transplantation. Therefore, it is likely that some of the ATN cases had subclinical ABMR, which could not be detected by routine histological assessment. Of note is that 4 of the 10 active ABMR patients exhibited ATN-like patterns.

The gene expressions in chronic active ABMR were higher than in those with early acute ABMR; the differences were obvious especially in the TI, as most of the patients exhibited higher Banff total inflammation scores. The expressions of the *SH2D1B*, *CX3CR1*, *IGHG1*, *IRF4*, *MS4A1*, *C5*, *CD46*, and *TGFB1* transcripts were higher in the TI of patients with chronic active ABMR; only the expression of the *CD14* gene predominated in the glomeruli. The upregulation of complement-associated gene transcripts in chronic ABMR was just recently reported (35). In our study, we showed the TI as a source of upregulated transcripts, including complement ones, in chronic active ABMR. The predominant sites of complement synthesis are renal tubules, but inflammatory cells can also contribute to complement production in inflamed tissues (36). The lower tubulointerstitial expression of *C5* from the samples obtained early after transplantation (ATN, active ABMR) may reflect tubular damage associated with early ischemic injury.

Interestingly, Cohen et al. (37) used LCM coupled with RT-qPCR in a small study and showed the upregulation of *CXCL10* expression in each renal compartment during severe rejection. In our study, the increased *CXCL10* expressions in both chronic active ABMR and active ABMR were observed mainly in the glomeruli. The *CXCL10* (*IP10*) chemokine was shown to be less specific, and its higher gene or protein expression reflects renal injury regardless of the origin. Similarly, *TGFB1* mRNA expression was shown to be expressed in both scarred or inflamed glomeruli and interstitium (38–40). Previously, we have shown that grafts with lower expressions of several transcripts, such as *TGFB1* or chemokines, were associated with poor kidney allograft outcomes (41). Similarly, in the present study, we observed the lowest *TGFB1* expression in grafts with active ABMR diagnosed early after transplantation. Nevertheless, our study was not powered to study the associations of gene transcripts with graft outcomes. Our observation of the upregulation of the monocyte marker *CD14* in the glomeruli of patients with chronic active ABMR is in line with previous studies that showed recruitment of monocytes into the glomerular compartment in ABMR (42). The observed compartment transcriptomic specificity reflects distinct cellular infiltrates that dominate either in the glomeruli or in the interstitium (26, 43).

The limitation of our study was the analysis of only several predefined transcripts due to the low RNA yield from laser-dissected glomeruli and TI, which does not allow either microarray RNA evaluation or RNA sequencing. Relative quantification of gene expression also limits the pathophysiological conclusions. Nevertheless, our study is the first systematically evaluating RNA transcripts from renal compartments in defined transplant pathology, while previous studies have mainly used immunohistochemistry for protein analyses or have focused on native kidneys. Clearly, the patient numbers are low to study outcomes, however high enough to see the differences in renal compartments. Of note is that the LCM method seems not suitable for routine assessments due to its extreme time requirements for sample preparation.

In conclusion, in this study, we showed distinct gene expression patterns in specific renal compartments reflecting cellular infiltration observed by conventional histology. In comparison with active ABMR, chronic active ABMR is associated with the increased transcripts of tubulointerstitial origin.

## Data Availability Statement

The original contributions presented in the study are included in the article/**Supplementary Material**. Further inquiries can be directed to the corresponding author.

## Ethics Statement

The studies involving human participants were reviewed and approved by the Ethics Committee of the Institute for Clinical and Experimental Medicine in Prague (No. A 13-02-01). The patients/participants provided their written informed consent to participate in this study.

## Author Contributions

AT and PM performed the research and wrote the manuscript. PH performed the research and statistical analysis. LV, ES, AS, MN, and MK performed the research. OV supervised the project and wrote the manuscript. All authors contributed to the article and approved the submitted version.

## Funding

This study was supported by the EU under ESIF, Operational Programme Research, Development and Education, CAMERA Project (CZ.02.2.69/0.0/0.0/17_050/0008096), and by the Ministry of Health of the Czech Republic under grants NV19-06-00031 and NU21-06-00021 and its conceptual development of research organizations (Institute for Clinical and Experimental Medicine-IKEM, IN 00023001).

## Conflict of Interest

The authors declare that the research was conducted in the absence of any commercial or financial relationships that could be construed as a potential conflict of interest.

## Publisher’s Note

All claims expressed in this article are solely those of the authors and do not necessarily represent those of their affiliated organizations, or those of the publisher, the editors and the reviewers. Any product that may be evaluated in this article, or claim that may be made by its manufacturer, is not guaranteed or endorsed by the publisher.
