# Supplementary material for: Chronic Active Antibody-Mediated Rejection Is Associated With the Upregulation of Interstitial But Not Glomerular Transcripts
Source: Front Immunol. 2021 Sep 20;12:729558. doi: 10.3389/fimmu.2021.729558 (PMC8488163; doi:10.3389/fimmu.2021.729558)

Supplementary Material

# Supplementary Tables

Table S1. Histological evaluation of kidney biopsies according to Banff 2019 classification

| **Histological scores**  **(0/1/2/3)** | **ATN** | **aABMR** | ***P*** | **Normal** | **caABMR** | ***P*** |
| --- | --- | --- | --- | --- | --- | --- |
| C4d | 11/0/0/0 | 1/0/1/8 | <0.001 | 12/0/0/0 | 3/4/1/1* | 0.004 |
| Glomerulitis (g) | 11/0/0/0 | 4/4/1/0* | 0.008 | 12/0/0/0 | 1/5/3/1 | <0.001 |
| Peritubular capillaritis score (ptc-s) | 11/0/0/0 | 7/2/0/0* | 0.099 | 12/0/0/0 | 2/1/4/0^†^ | 0.001 |
| Peritubular capillaritis quality (ptc-q) | 11/0/0/0 | 7/2/0/0* | 0.099 | 12/0/0/0 | 2/0/5/0^†^ | <0.001 |
| Peritubular capillaritis extent (ptc-e) | 11/0/0/0 | 7/2/0/0* | 0.099 | 12/0/0/0 | 2/2/3/0^†^ | 0.001 |
| Glomerular capillaries double contours (cg) | 11/0/0/0 | 9/0/0/0* | 1.000 | 12/0/0/0 | 0/1/5/4 | <0.001 |
| Mesangial matrix increase (mm) | 11/0/0/0 | 9/0/0/0* | 1.000 | 12/0/0/0 | 1/5/3/1 | <0.001 |
| Arteritis (v) | 10/0/0/0 | 8/1/0/0* | 0.279 | 12/0/0/0 | 7/2/0/0* | 0.086 |
| Interstitial inflammation (i) | 11/0/0/0 | 9/0/0/0* | 1.000 | 12/0/0/0 | 8/0/0/0^‡^ | 1.000 |
| Tubulitis (t) | 11/0/0/0 | 9/0/0/0* | 1.000 | 12/0/0/0 | 8/0/0/0^‡^ | 1.000 |
| Interstitial inflammation in total parenchyma (ti) | 11/0/0/0 | 7/2/0/0* | 0.099 | 11/1/0/0 | 1/3/2/2^‡^ | <0.001 |
| Fibrous intimal thickening (cv) | 0/3/3/4 | 0/5/2/2* | 0.268 | 2/5/4/1 | 0/2/4/3* | 0.048 |
| Arteriolar hyaline thickening (ah) | 1/7/1/2 | 1/3/4/1* | 0.621 | 1/5/4/2 | 0/2/1/7 | 0.025 |
| Alternate arteriolar hyaline thickening (aah) | 7/4/0/0 | 7/1/0/1* | 0.807 | 7/4/0/1 | 2/0/1/4^†^ | 0.017 |
| Interstitial fibrosis (ci) | 0/10/1/0 | 2/7/0/0* | 0.070 | 2/10/0/0 | 0/1/6/1^‡^ | <0.001 |
| Tubular atrophy (ct) | 0/10/1/0 | 2/7/0/0* | 0.070 | 2/10/0/0 | 0/1/6/1^‡^ | <0.001 |

Pearson’s chi-squared test.

* data from 1 sample was not available

^†^ data from 3 samples were not available

^‡^ data from 2 samples were not available

Table S2. Genes evaluated by RT-qPCR.

| **Gene** | **Coding protein** | **Main source.**  **Significance** | **Assay ID** |
| --- | --- | --- | --- |
| *IFNG* (Interferon-gamma) | IFNG | NK-cells, T-cells.  TCMR-associated (1) | Hs00989291_m1 |
| *GNLY* (Granulysin) | GNLY | NK-cells, T-cells.  DSA selective, ABMR-associated (2) | Hs01120727_m1 |
| *CD14* (Monocyte Differentiation Antigen CD14) | CD14 | Mon, MC.  ABMR-associated (3) | Hs00169122_g1 |
| *SH2D1B* (SH2 Domain-Containing Protein 1B) | SH2D1B | NK-cells, T-cells.  DSA selective, ABMR-selective (4) | Hs01592483_m1 |
| *CX3CR1* (C-X3-C Motif Chemokine Receptor 1) | CX3CR1 | NK-cells, Mon, MC.  DSA selective, ABMR-selective (2) | Hs00365842_m1 |
| *IGHG1* (Immunoglobulin IgG1 Heavy Chain Constant Region) | IGHG1 | B-cells, plasma-cells.  ABMR-associated (5) | Hs00378340_m1 |
| *IRF4* (Interferon Regulatory Factor 4) | IRF4 | B-cells, T-cells, Mon, MC  ABMR-associated (5) | Hs00180031_m1 |
| *TNFRSF17 (*TNF Receptor Superfamily Member 17) | TNFRSF17 | B-cells, plasma-cells.  ABMR-associated (5) | Hs03045080_m1 |
| *MS4A1* (Membrane Spanning 4-Domains A1) | CD20 | B-cells.  ABMR-associated (6) | Hs00174849_m1 |
| *TGFB1* (Transforming Growth Factor b1) | TGFB1 | Ubiquitous.  rejection-associated (7) | Hs00998133_m1 |
| *C5* (Complement Component C5) | C5 | Mon, MC, DC, EC, fibroblasts, muscle cells.  ABMR-associated (8) | Hs01004342_m1 |
| *CD46* (Membrane Cofactor Protein) | CD46 (MCP) | Ubiquitous.  Complement regulation.  ABMR-associated (9) | Hs00611257_m1 |
| *CXCL10* (C-X-C Motif Chemokine Ligand 10) | CXCL10 | Leukocytes, TEC, EC, fibroblasts.  IFNG-inducible, AMBR and rejection associated (10) | Hs00171042_m1 |
| *HAVCR1* (Hepatitis A Virus Cellular Receptor 1) | KIM1 (Kidney injury molecule 1) | TEC.  Acute Kidney Injury-associated (11) | Hs00273334_m1 |
| **Controls** | | | |
| *NPHS2* (NPHS2 Stomatin Family Member) | Podocin | podocytes | Hs00387817_m1 |
| *GAPDH* (Glyceraldehyde-3-Phosphate Dehydrogenase) | GAPDH | ubiquitous | Hs99999905_m1 |

Abbreviations: ABMR: antibody-mediated rejection, DC: dendritic cells, DSA: donor-specific antibodies, EC: endothelial cells, Mon: monocytes, MC: macrophages, TEC: tubular epithelial cells.

1. Venner, J. M., Hidalgo, L. G., Famulski, K. S., Chang, J., Halloran, P. F. (2015). The molecular landscape of antibody-mediated kidney transplant rejection: evidence for NK involvement through CD16a Fc receptors. Am J Transplant. 15, 1336-1348.

2. Sellares, J., Reeve, J., Loupy, A., Mengel, M., Sis, B., Skene, A., et al. (2013). Molecular diagnosis of antibody-mediated rejection in human kidney transplants. Am J Transplant. 13, 971-983.

3. Magil, A. B. (2009). Monocytes/macrophages in renal allograft rejection. Transplant Rev (Orlando). 23, 199-208.

4. Hidalgo, L. G., Sis, B., Sellares, J., Campbell, P. M., Mengel, M., Einecke, G., et al. (2010). NK cell transcripts and NK cells in kidney biopsies from patients with donor-specific antibodies: evidence for NK cell involvement in antibody-mediated rejection. Am J Transplant. 10, 1812-1822.

5. Einecke, G., Reeve, J., Mengel, M., Sis, B., Bunnag, S., Mueller, T. F., et al. (2008). Expression of B cell and immunoglobulin transcripts is a feature of inflammation in late allografts. Am J Transplant. 8, 1434-1443.

6. Heidt, S., Vergunst, M., Anholts, J. D. H., Swings, G., Gielis, E. M. J., Groeneweg, K. E., et al. (2019). Presence of intragraft B cells during acute renal allograft rejection is accompanied by changes in peripheral blood B cell subsets. Clin Exp Immunol. 196, 403-414.

7. Elster, E. A., Hawksworth, J. S., Cheng, O., Leeser, D. B., Ring, M., Tadaki, D. K., et al. (2010). Probabilistic (Bayesian) modeling of gene expression in transplant glomerulopathy. J Mol Diagn. 12, 653-663.

8. Reis, E. S., Barbuto, J. A., Isaac, L. (2006). Human monocyte-derived dendritic cells are a source of several complement proteins. Inflamm Res. 55, 179-184.

9. Hruba, P., Krejcik, Z., Stranecky, V., Maluskova, J., Slatinska, J., Gueler, F., et al. (2019). Molecular Patterns Discriminate Accommodation and Subclinical Antibody-mediated Rejection in Kidney Transplantation. Transplantation. 103, 909-917.

10. Halloran, P. F., Venner, J. M., Madill-Thomsen, K. S., Einecke, G., Parkes, M. D., Hidalgo, L. G., et al. (2018). Review: The transcripts associated with organ allograft rejection. Am J Transplant. 18, 785-795.

11. Famulski, K. S., de Freitas, D. G., Kreepala, C., Chang, J., Sellares, J., Sis, B., et al. (2012). Molecular phenotypes of acute kidney injury in kidney transplants. J Am Soc Nephrol. 23, 948-958.

Table S3. Correlation of transcript expression with selected histological scores

|  |  | Glomerulitis (g) | Peritubular capillaritis score (ptc-s) | Microvascular inflammation (MVI) | Glomerular capillaries double contours (cg) | Interstitial inflammation in total parenchyma (ti) | Interstitial fibrosis (ci) |
| --- | --- | --- | --- | --- | --- | --- | --- |
| IFNG | glomeruli | ** | ** | *** | * | NS | NS |
|  | TI | * | NS | ** | ** | *** | NS |
| GNLY | glomeruli | ** | *** | ** | ** | NS | NS |
|  | TI | ** | *** | *** | ** | ** | NS |
| CD14 | glomeruli | * | NS | NS | *** | NS | * |
|  | TI | NS | NS | NS | ** | * | NS |
| SH2D1B | glomeruli | NS | NS | NS | NS | NS | NS |
|  | TI | ** | *** | ** | *** | * | * |
| CX3CR1 | glomeruli | * | *** | * | * | NS | NS |
|  | TI | ** | *** | ** | *** | *** | NS |
| IGHG1 | glomeruli | - | - | - | - | - | NS |
|  | TI | * | NS | NS | ** | NS | NS |
| IRF4 | glomeruli | NS | * | * | NS | NS | NS |
|  | TI | *** | ** | *** | *** | *** | * |
| MS4A1 | glomeruli | NS | NS | NS | NS | NS | NS |
|  | TI | NS | NS | NS | * | ** | NS |
| TGFB1 | glomeruli | *** | NS | ** | ** | * | NS |
|  | TI | ** | ** | ** | *** | *** | NS |
| C5 | glomeruli | NS | NS | NS | NS | NS | NS |
|  | TI | NS | NS | NS | * | * | NS |
| CD46 | glomeruli | NS | NS | NS | NS | NS | NS |
|  | TI | NS | NS | NS | NS | NS | NS |
| CXCL10 | glomeruli | ** | ** | *** | ** | * | NS |
|  | TI | * | *** | * | * | NS | NS |

Spearman’s rank correlation test.

* P < 0.05

** P < 0.01

*** P < 0.001

NS not significant

# Supplementary Figures

**Supplementary Figure S1.** The expression of glomeruli-specific podocin (NPSH2) transcript in glomeruli and tubulointerstitium as a control of sampling accuracy.

**
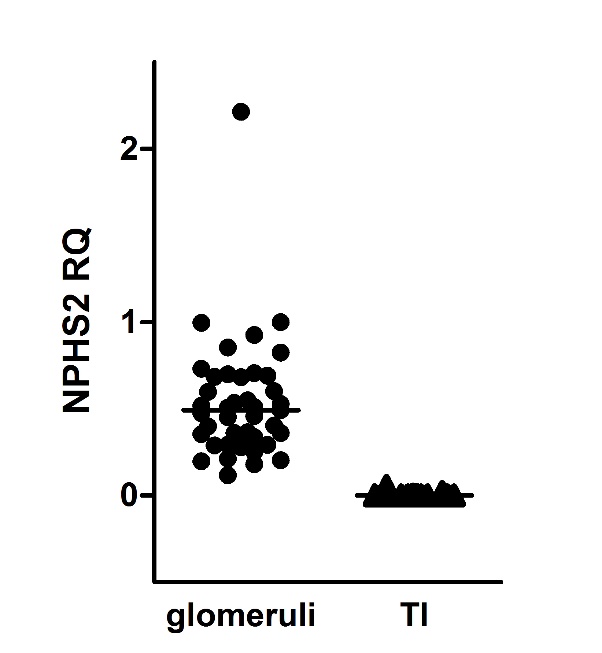
**

**Supplementary Figure S2.** Differences in gene expression (log2 FC) between acute tubular necrosis (ATN) and active ABMR (aABMR), normal 3M protocol biopsies (3M normal) and chronic active ABMR (caABMR) and aABMR and caABMR in glomeruli (dark marks) and tubulointerstitium (empty marks). Genes up-regulated in ABMR in glomeruli or both glomeruli and tubulointerstitium (**A**). Genes up-regulated in ABMR in tubulointerstitium (**B**).

Differences are calculated by Mann-Whitney test.

**
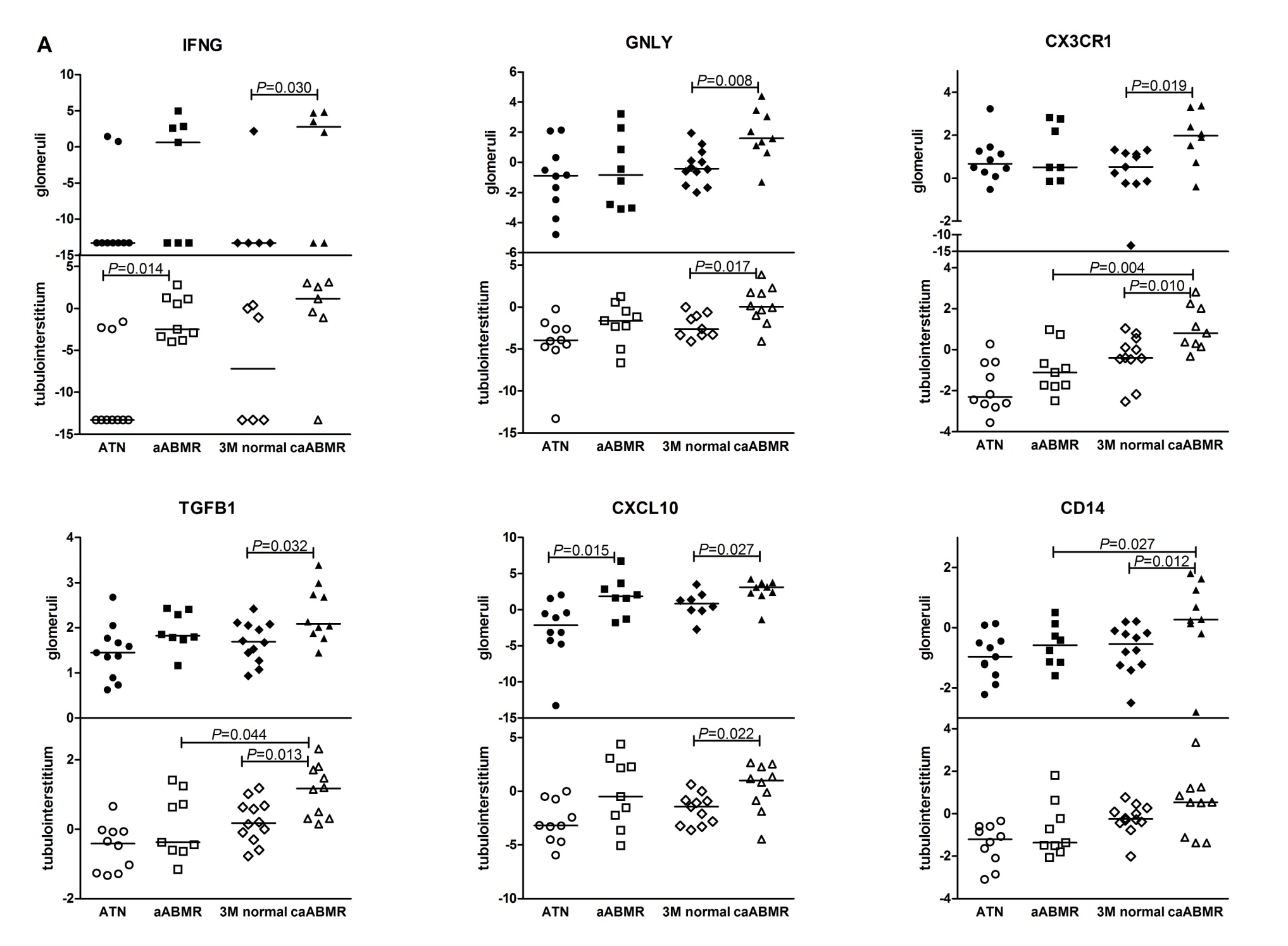

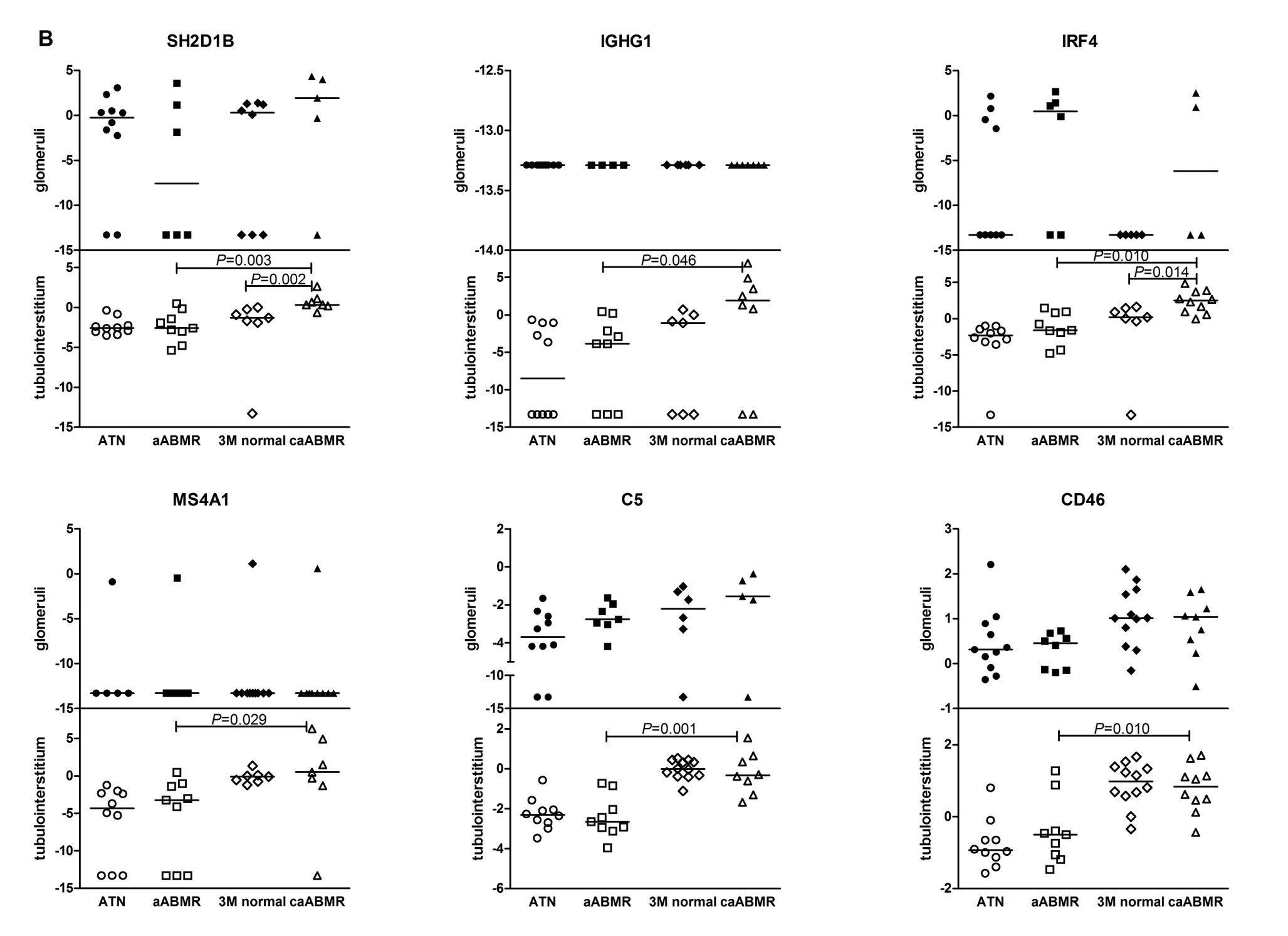
**

**Supplementary Figure S3.** Differences in gene expression (log2 FC) between glomeruli (G, dark bars) and tubulointerstitium (TI, empty bars) in the groups with: A) acute tubular necrosis (ATN, n=11), and B) normal 3M protocol biopsies (3M normal, n=12). Boxes extends from the 25th to 75th percentiles, lines in the middle of the box shows median and whiskers indicate the highest and lowest value within inner fences (Tukey whiskers).

Differences are calculated by Mann-Whitney test.


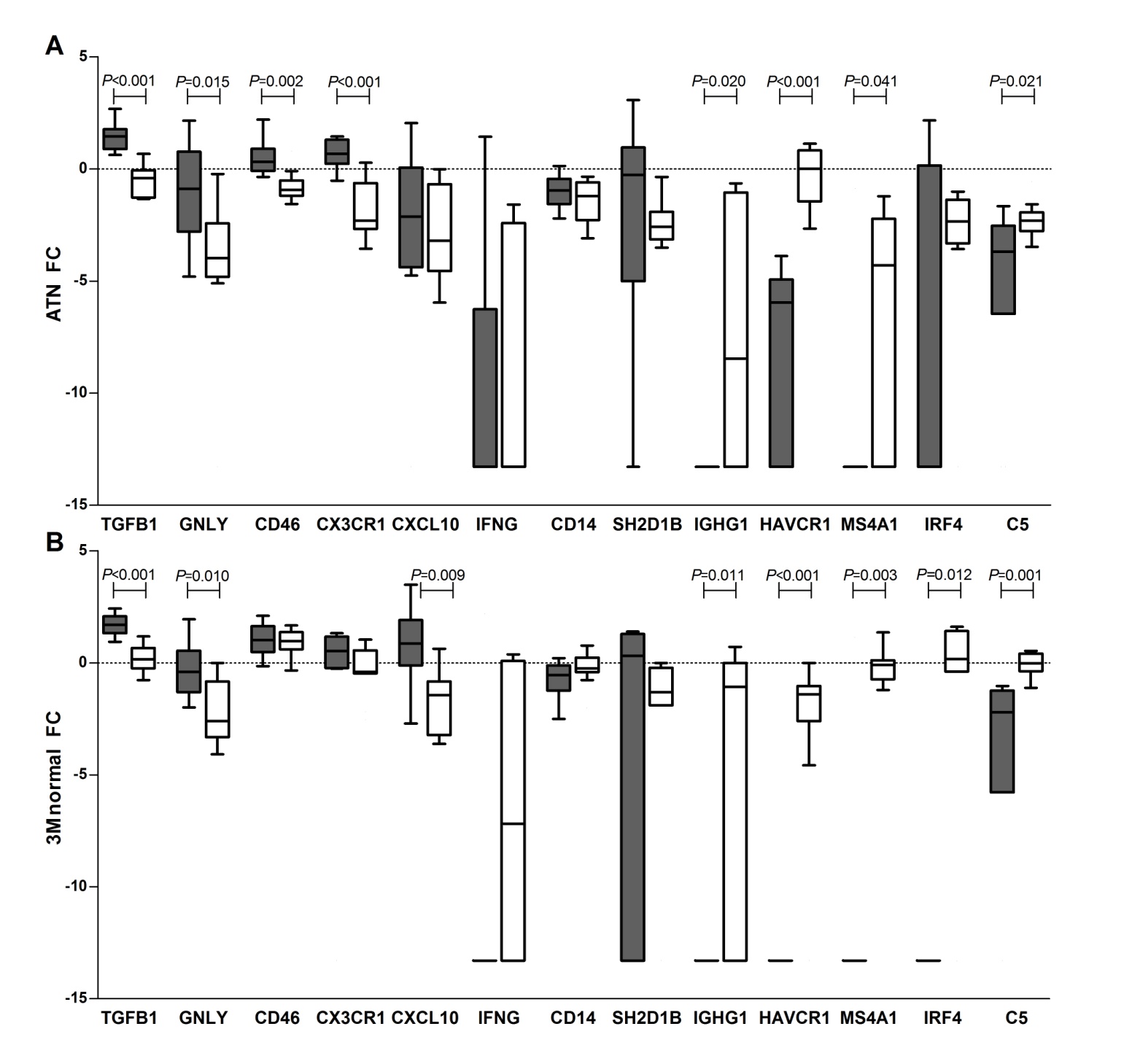

Supplement: Supplementary file 1 [file DataSheet_1.docx]
